# One Health activities to reinforce intersectoral coordination at local levels in India

**DOI:** 10.3389/fpubh.2023.1041447

**Published:** 2023-03-07

**Authors:** Jessica Taaffe, Rajnish Sharma, Aravindh Babu R. Parthiban, Jaswinder Singh, Paviter Kaur, Balbir B. Singh, Jatinder P. S. Gill, Dhinakar Raj Gopal, Navneet K. Dhand, Falgunee K. Parekh

**Affiliations:** ^1^EpiPointe, Cary, NC, United States; ^2^Centre for One Health, College of Veterinary Science, Guru Angad Dev Veterinary and Animal Sciences University, Ludhiana, Punjab, India; ^3^Translational Research Platform for Veterinary Biologicals, Centre for Animal Health Studies, Tamil Nadu Veterinary and Animal Sciences University, Chennai, Tamil Nadu, India; ^4^Department of Veterinary and Animal Husbandry Extension Education, College of Veterinary Science, Guru Angad Dev Veterinary and Animal Sciences University, Ludhiana, Punjab, India; ^5^Department of Veterinary Microbiology, College of Veterinary Science, Guru Angad Dev Veterinary and Animal Sciences University, Ludhiana, Punjab, India; ^6^Sydney School of Veterinary Science, The University of Sydney, Sydney, NSW, Australia

**Keywords:** One Health, India, intersectoral collaboration, zoonotic diseases, emerging infectious diseases

## Abstract

India's dense human and animal populations, agricultural economy, changing environment, and social dynamics support conditions for emergence/re-emergence of zoonotic diseases that necessitate a One Health (OH) approach for control. In addition to OH national level frameworks, effective OH driven strategies that promote local intersectoral coordination and collaboration are needed to truly address zoonotic diseases in India. We conducted a literature review to assess the landscape of OH activities at local levels in India that featured intersectoral coordination and collaboration and supplemented it with our own experience conducting OH related activities with local partners. We identified key themes and examples in local OH activities. Our landscape assessment demonstrated that intersectoral collaboration primarily occurs through specific research activities and during outbreaks, however, there is limited formal coordination among veterinary, medical, and environmental professionals on the day-to-day prevention and detection of zoonotic diseases at district/sub-district levels in India. Examples of local OH driven intersectoral coordination include the essential role of veterinarians in COVID-19 diagnostics, testing of human samples in veterinary labs for *Brucella* and leptospirosis in Punjab and Tamil Nadu, respectively, and implementation of OH education targeted to school children and farmers in rural communities. There is an opportunity to strengthen local intersectoral coordination between animal, human and environmental health sectors by building on these activities and formalizing the existing collaborative networks. As India moves forward with broad OH initiatives, OH networks and experience at the local level from previous or ongoing activities can support implementation from the ground up.

## Introduction

One Health (OH) is a framework for a transdisciplinary and cross-sectoral approach to address complex problems at the intersection of the environment, people, plants, and animals. The OH approach saves human and animal lives and is economical due to the efficient use of resources, for example, infrastructure, finances, personnel, and timely action (1, 2). In addition to critical global health concerns like food safety and antimicrobial resistance (AMR), the OH approach is essential to address zoonotic diseases, as the interactions at the human-animal-environment interface directly influence the epidemiology of these diseases. The recent COVID-19 pandemic and global monkeypox public health emergency highlight the ongoing global threat of emerging and re-emerging zoonotic diseases, with calls for using a OH approach to prepare for and address the next infectious disease threat (3, 4). With 75% of emerging infections having a non-human animal origin (5), the next global infectious disease emergency will likely require a OH approach.

The drivers of infectious diseases' emergence/re-emergence include increasing human population, urbanization, climate change, land use change, intensive farming practices, deforestation, and exploitation of wildlife. Human population density, land area and the human development index at the country level are associated with human, emerging and zoonotic pathogen diversity (6). A One Health approach is important for India, as it is considered one of the probable global “hotspots” for emerging and re-emerging zoonoses (7) due to its enormous human and animal populations and complex agricultural economy combined with rapid socio-ecological, environmental and climactic changes. It accounts for 35% of the global burden of rabies (8), with ~20,000 human deaths annually. Economic loss due to the brucellosis burden in livestock in India was estimated to be 3.4 billion USD (9). Along with four nations, India contributes to 83% of cases of Kala-azar (visceral leishmaniasis) globally (10). Moreover, highly pathogenic zoonotic diseases like Nipah virus and Crimean-Congo Hemorrhagic Fever (CCHF) virus have emerged in India in the past two decades.

A key component of OH is enhancing interactions and collaborations among diverse stakeholders, not only at the international and national levels, but also at the local level. It is particularly important in India, where most of the human population continues to live in rural areas and have daily interactions with livestock and wildlife. In many areas, animals, including cattle, dogs, and pigs, roam freely in the streets. Environmental and climate factors, such as temperature, humidity, population density, deforestation, pollutants, change in land use etc., may lead to the emergence/re-emergence and increased transmission of zoonotic pathogens. In these areas and communities with high human-animal-environmental interaction, effective OH driven strategies that promote local intersectoral coordination and collaboration are needed to truly address zoonotic diseases in India.

**Figure 1:**
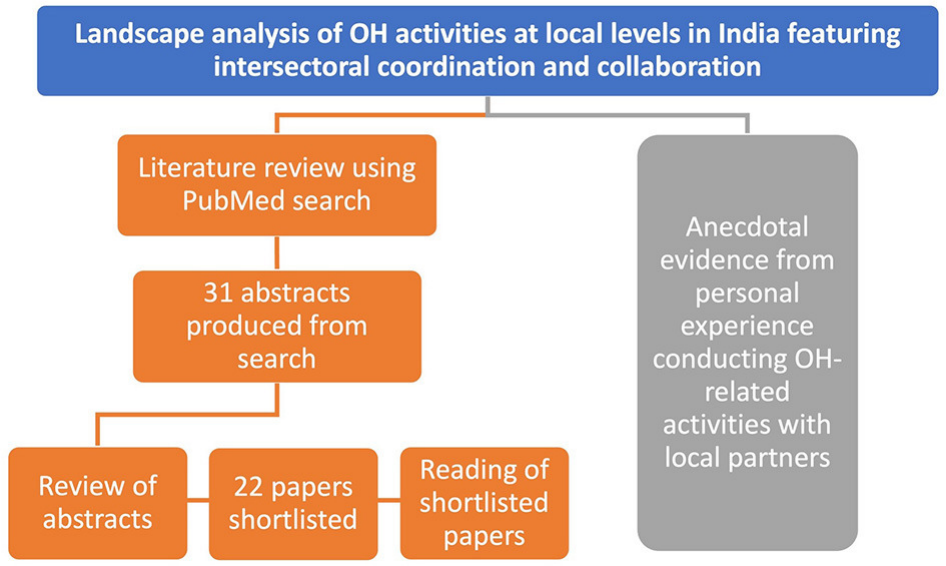
Overview of landscape analysis approach to evaluate OH activities at local levels in India featuring intersectoral coordination and collaboration. A literature review using PubMed search produced 31 abstracts based on search terms. These abstracts were reviewed, and 22 of them were shortlisted for in-depth reading and evaluation of these publications by co-authors. In parallel, examples of local intersectoral coordination and collaboration on OH activities were identified through personal experience conducting and knowledge of relevant activities with local partners.

While there is support for OH frameworks in India, particularly at the national level, OH-related efforts at local levels (ex. district, sub-district, city, Tehsil or taluk, and village levels) are critical, especially for addressing zoonotic and emerging diseases at the level at which they start. To assess the landscape of OH activities at local levels in India featuring intersectoral coordination and collaboration, we conducted a landscape review of the literature and supplemented it with our own experience conducting OH-related activities with local partners (Figure 1). We conducted a PubMed search including keywords “One Health[Title]” and “India[Title]” on 8 July 2022, which returned 31 relevant published papers. Two co-authors (JT and ND) reviewed 31 abstracts, excluded two that were corrections to already included papers, and shortlisted 22 full-length papers for in-depth reading. Eight co-authors were asked to read these papers and note the following information: (a) Was there intersectoral coordination? If yes, which sectors were involved? (b) What were the findings and overall paper focus? (c) Who was involved at the local level? (d) Was there community involvement? This information enabled us to identify key overarching themes and examples in local OH activities in India.

## Findings

Our review of OH in India literature returned publications falling under the following categories: (1) Studies describing public health, epidemiological, and laboratory research, including reviews, (2) Policy or social sciences research or analysis of Indian OH frameworks, and (3) Case studies of OH relevant programs or activities (Table 1). Our landscape assessment demonstrated that OH intersectoral collaboration primarily occurs through specific research activities, during outbreaks, and for community outreach efforts. However, there is limited formal coordination among veterinary, medical, and environmental professionals on the day-to-day prevention and detection of zoonotic diseases at district/sub-district levels in India. Here we describe examples of OH activities featuring local coordination and collaboration from the published literature and our own experiences implementing them.

**Table 1:** Summary of One Health in India literature review.

**References**	**Year**	**Category**	**Author-specified keywords**	**Key descriptors**
Chaudhari et al. (11)	2021	Laboratory and/or epidemiological research	Brucellosis; One Health; Listeriosis; Scrub typhus; Surveillance; Zoonotic diseases	Research collaboration, Zoonotic disease surveillance
Das et al. (12)	2020	Laboratory and/or epidemiological research	India; One Health; Antibiotics resistance; Bacterial infection; Drug prescriptions; Sentinel surveillance	Surveillance; AMR; Research collaboration
Sack et al. (13)	2021	Laboratory and/or epidemiological research	Not specified	Epidemiological investigation; Research collaboration; Environmental sampling
Asaaga et al. (14)	2021	Policy/Social science	Cross-sectoral convergence; Emerging infectious disease; Health system; India; One Health; Zoonoses	Operationalisation/implementation facilitators and barriers; Zoonosis control
Perez et al. (15)	2020	Case study; Policy	Avian influenza; Flood management; One Health action; Rabies	Operationalisation/implementation; Zoonosis control
Yasobant et al. (16)	2021	Case study; Policy/Social science	ASHA; CHW; India; Motivation; OHA; One Health	Implementation; Community engagement
Jani et al. (17)	2021	Laboratory and/or epidemiological research	Antimicrobial resistance, Mass gatherings, AMR containment policy, One Health approach	AMR; Review
Chattu et al. (18)	2018	Case study; Policy	Global health security; Kerala; Nipah virus; One Health; Pteropus bat species; Paramyxovirus	Zoonotic disease outbreak; Research collaboration
Rajagopal et al. (19)	2021	Laboratory and/or epidemiological research	Escherichia coli; India; One Health; Antimicrobial resistance; Community-acquired	AMR; Review
Yasobant et al. (20)	2021	Public health research; Social science	Health system contact; India; One Health; Community awareness; Zoonotic diseases	Community awareness investigation
Dasgupta et al. (21)	2021	Policy	Intersectoral approach; One Health committee; Leadership; Strategic goals; Zoonoses	Operationalisation/implementation
Yasobant et al. (22)	2020	Case study; Policy/Social science	India; One Health; Actors; Health system; Intersectoral collaboration	Operationalisation; Outbreak
Reddy et al. (23)	2021	Policy	Not specified	Operationalisation; Outbreak
Yasobant et al. (24)	2019	Policy	Health system; India; Initiatives; One Health collaboration; Strategies	Zoonoses; Vaccination; Education
Yasobant et al. (25)	2020	Policy	Coronavirus disease 2019; India; One Health; One Health surveillance	Surveillance; Outbreak
Lindahl et al. (26)	2020	Policy	Brucella; Brucellosis; India; Livestock; Public health	Zoonotic disease control
Fitzpatrick et al. (27)	2016	Laboratory and/or epidemiological research	Cost-effectiveness; Mathematical modeling; Rabies; Sterilization; Vaccination	Zoonotic disease control; Cost-effectiveness; Economic analysis
Yasobant et al. (28)	2018	Policy/Social science	One Health; Systems thinking; Health systems; Prevention and control; Zoonotic diseases	Zoonotic disease control
Yasobant et al. (29)	2019	Policy/Social science	Not specified	Disease prioritization; Zoonotic diseases; Stakeholder engagement
Weiss et al. (30)	2021	Policy	Not specified	Perspective piece; One Health in India
Chatterjee et al. (31)	2016	Policy	Emerging infectious diseases; Health policy; Intersectoral coordination; Multidisciplinary; One Health; Transdisciplinary; Zoonoses	Perspective piece; One Health in India
Yasobant et al. (32)	2021	Case study, Policy	Intersectoral collaboration; One Health; Operationalization; Health system; India	Zoonotic diseases; Operationalization
Mansingh et al. (33)	2021	Policy; Public health research	Anthrax; FDG; One Health; Endemic regions; Surveillance; Zoonotic disease	Zoonotic diseases
Mourya et al. (34)	2021	Case study	Crimean-Congo haemorrhagic fever; India; Kyasanur forest disease; One Heath; Tick-borne; Zoonotic disease	Outbreak
Stålsby et al. (35)	2015	Laboratory and/or epidemiological research	One Health; Health seeking behavior; Antibiotic prescribing; Formal and informal health care providers; Escherichia coli in stools of children and water; Antibiotic resistance; Molecular basis of resistance	AMR
Murhekar et al. (36)	2021	Case study, Laboratory and/or epidemiological research	Acute encephalitis syndrome; One Health; Acute febrile illness; Scrub typhus; Vector	Zoonotic disease surveillance; Zoonotic disease control; Outbreak
Prejit et al. (37)	2022	Case study; Public health research	Evaluation; Integrated surveillance; KFD virus; One Health; Wayanad; Zoonoses	Zoonotic disease surveillance; Zoonotic disease control; Outbreak
Gibson et al. (38)	2022	Case study; Public health research	Developing world; Epidemiology; Viral epidemiology; Viral genetics	Zoonotic disease surveillance; Zoonotic disease control; Economic analysis
Abbas et al. (39)	2011	Case study; Public health research	Rabies, Zoonosis, India, One Health, Health policy, Communicable disease	Zoonotic disease control

Twenty-nine papers from a search on One Health in India are described by their year of publication, category, author-specified keywords, and other key descriptors. Author-specified keywords were provided by each publication's authors. Key descriptors were derived in our abstract and/or full paper review of each publication.

### Research and laboratory collaborations

Many OH activities in India are public health research-based, largely focusing on the etiology and surveillance of zoonotic diseases and sometimes AMR. Intersectoral collaboration happens primarily between human health and animal health scientists, though AMR studies may also involve environmental sampling and/or participation from the environmental sector. In a systematic review of studies from 18 states examining antimicrobial-resistant *E. coli* across India, only 10% (4 out of 38) conducted interdisciplinary sampling—defined as sampling from a combination of human, animal, or environmental sources (19). The research studies often feature a multidisciplinary approach, combining social/epidemiological surveys with laboratory-based and/or economic investigations (Table 1). For example, a transdisciplinary team of human and animal health researchers conducted several studies to identify *Orientia tsutsugamushi*, the causative agent of scrub typhus, as the major etiology of Acute Encephalitis Syndrome (AES) outbreaks in Gorakhpur region, India (36). These studies led to a better understanding of AES transmission in the region and recommendations for its control.

Veterinary institutes are essential partners in OH research and laboratory diagnostics. In early 2020, Tamil Nadu Veterinary and Animal Sciences University (TANUVAS) signed a MoU with The Tamil Nadu Dr MGR Medical University (TNMGRMU) for conducting joint academic and research activities, including an animal trial for a SARS-CoV2 vaccine candidate developed by TNMGRMU. TANUVAS has also performed *in vitro* testing for some plant-derived SARS-CoV-2 therapeutic compounds (A.B.R. Parthiban, personal communication, August 2022). Guru Angad Dev Veterinary and Animal Sciences University (GADVASU) receives samples from Dayanand Medical College and Hospital, Ludhiana for testing of *Brucella* spp. Likewise, TANUVAS initiated diagnostic testing for human leptospirosis about two decades ago using a microscopic agglutination test and dark field microscopy, gold standard confirmatory tests for leptospirosis. TANUVAS maintains leptospirosis reference cultures (serovars) for these testing. Even today, TANUVAS remains the only laboratory to offer these tests in tandem in the southern states of India. In contrast, commercial testing laboratories offer only IgM/ELISA-based screening tests. The testing results on leptospirosis are periodically shared with the Tamil Nadu State Public Health Department (A.B.R. Parthiban, personal communication, August 2022). Moreover, the Ministry of Health and Family Welfare (MoHFW) designated two veterinary labs—National Research Center on Equines, Hissar and Central Military Veterinary Laboratory), Meerut—as reference laboratories for the diagnosis of glanders in humans (40).

### Disease outbreaks

Intersectoral interactions at the village/sub-district level have occurred most frequently during disease outbreaks and have been observed across the country (22, 36, 41, 42). These interactions often involve OH coordination and response between stakeholders at national, state, and district levels, usually with a top-down approach that directs, initiates, and supports response from lower-level stakeholders. For example, the Indian Council of Medical Research (ICMR)—National Institute of Virology, Pune acted at the local level to build laboratory capacity and develop laboratory networks for quick diagnosis of emerging infectious diseases through their activities managing Kyasanur Forest disease and CCHF outbreaks in India (34). Similarly, an intersectoral collaboration featuring the MoHFW, Directorate of Health Research, Indian Council of Agricultural Research (ICAR), State Health Department, State Animal Husbandry, and District Administration, during a 2018 Nipah virus outbreak in Kerala led to zero spread and no mortality in a subsequent outbreak the following year (42). Currently, a OH approach at multiple levels is being utilized to control anthrax in several villages of a tribal district of Odisha (41); several stakeholders are involved: clinical service providers, program managers and health workers (health care sector), veterinary doctors, livestock inspectors, forest guards (animals care sector) and service utilizer clients, local governance members, non-governmental organizations (NGOs), self-help groups, cattle owners/gatherers, and village residents (community) (41).

Animal health professionals recently supported their human health counterparts during the COVID-19 pandemic. In the Haryana state, veterinarians were deployed in the isolation wards of village hospitals (43). Veterinarians were not only engaged from the animal husbandry departments for helping medical staff during this pandemic but also from other departments, such as veterinary corps personnel from the Armed forces. They were deployed through the operation “CO-JEET (Victory over COVID; Jeet means Victory)” to support overstretched medical personnel (44) and were involved in several activities, including setting up of COVID facilities (45).

Enhanced intersectoral coordination in laboratory diagnostics also featured prominently during the COVID pandemic. For example, scientists from several veterinary schools and agencies supported their medical counterparts during the beginning of the pandemic in India (46). In April 2020, a team of 10 researchers from GADVASU joined the team of medical doctors at the Government Medical colleges, Amritsar and Patiala, providing two Real-Time PCR testing machines and training on how to use these machines for diagnostic testing (47). Veterinarians in many parts of the country also stepped in to perform temperature screens, COVID tests, and collect patient samples. Even more, in August 2020, a COVID-19 Testing laboratory was established at GADVASU, playing a critical role in diagnosing COVID-19 in Punjab. Within 6 months, the lab tested more than 100,000 samples (48). Another example of such intersectoral coordination is when TANUVAS partnered with Kings Institute of Preventive Medicine and Council of Scientific and Industrial Research-Center for Cellular and Molecular Biology from April-May 2020 to sequence 21 complete genomes of SARS-CoV2. These were the first complete genome sequences of SARS-CoV2 circulating in Tamil Nadu to be submitted to the Global Initiative on Sharing Avian Influenza Data's Epicov (GISAID's EpiCoV) database (EPI_SET_220907pk, https://doi.org/10.55876/gis8.220907pk).

### Community outreach and engagement

The OH-related intersectoral coordination and collaboration must involve communities to improve and sustain the prevention, detection and control of zoonotic and emerging pathogens, particularly in high-risk areas. Beyond medical and veterinary personnel, local leaders, community health workers, and NGOs have been instrumental in outbreak response. Collaboration between several stakeholders, for example, the village sarpanch (head of village-level constitutional body), community health officers, auxiliary nursing midwives, and accredited social health activists (ASHA), enabled overcoming the COVID vaccine hesitancy in a village of Punjab (49). Similarly, grassroots NGOs that work closely with tribal communities in the Nilgiri district of Tamil Nadu were recruited to address vaccine hesitancy in these communities (50).

Another way the OH approach is implemented in communities is through community outreach and education. GADVASU has established the Center for OH that engages with the community to educate them about zoonotic diseases, AMR, food safety, and biosecurity measures in various training and education programs. Under ICAR's Farmers FIRST Programme, a multi-disciplinary team comprising of animal scientists, an agronomist, and a vegetable expert from GADVASU and Punjab Agricultural University worked closely with sarpanches of five villages to educate and help them in quality crop, vegetable, and fodder production, clean milk production, vector control (flies and ticks), mastitis detection, and in the construction of foot baths at farm gates for stronger biosecurity (J. Singh, personal communication, August 2022). In 2016, GADVASU and the State health department organized an awareness camp at the livestock farmers' fair to inform the visitors about symptoms and preventive measures for prevalent diseases such as brucellosis. They also provided free brucellosis testing for farmers, testing 125 samples (51). Community outreach has also been extended to school children, educating them about rabies, food safety, hygienic practices and AMR (52–55). Aside from in-person events and training, the university disseminates knowledge on OH topics through print (books, magazines), digital tools (apps, YouTube channels, Facebook, etc.), and mass media (newspaper, radio, and television) (J. Singh, personal communication, August 2022).

## Discussion

The OH approach to addressing zoonotic diseases amid increasing enablers like globalization, urbanization, climate change, etc., is critical globally and especially for India. As evidenced by the number of initiatives and publications describing initiatives or suggested frameworks for OH implementation identified through our literature review, there appears to be excellent support for implementing OH in India.

Addressing zoonotic diseases in India is complicated because of the complex and non-integrated infrastructure of its health (animal and human) and environmental agencies. Zoonotic diseases in humans, domestic animals and wild animals fall under the purview of several ministries, including the MoHFW, the Ministry of Agriculture, and the Wildlife Institute of India, respectively (56). Furthermore, issues relating to forests and climate change come under the purview of the Ministry of Environment, Forest and Climate Change. These different ministries often have focused and non-overlapping objectives and priorities, challenging coordinated operationalization of the OH approach for zoonotic diseases in India. Moreover, while overarching policies, regulations, and outbreak response are managed from the federal level, animal and human health initiatives are implemented by state governments, which set their own priorities based on the local context and budget. Central frameworks, dedicated funding, or data-sharing mechanisms to implement OH across sectors are lacking because of this governmental structure. Other factors such as infrastructure, zoonotic disease knowledge, training, response capacity, inter-sector politics, and disparate human and animal disease reporting systems make implementing OH in India challenging from local to central levels (14).

Efforts to improve the implementation of OH through enhanced cross-sectoral coordination and collaboration at the national and state level are ongoing (14). The Roadmap to Combat Zoonoses in India was launched in 2008 to facilitate coordination among human, animal and wildlife health sectors, and to identify priority research areas for zoonoses (56). In 2017, India prepared a National Action Plan on AMR, taking the OH approach involving human, animal and environmental sectors (10). Additionally, a national-level OH Consortium led by the Department of Biotechnology -National Institute of Animal Biotechnology, Hyderabad has recently been established, consisting of 27 organizations, including several medical, veterinary, and wildlife agencies (57), and zoonotic research toward the establishment of a National Institute of OH in India has been jointly supported by the ICMR and the ICAR (11).

The objective of our literature review was to identify examples of local OH activities and coordination. We focused primarily on published peer-reviewed literature and did not include gray literature in our search methods, which might have limited the activities we found reported. In general, we found that most of the literature focusing on OH in India could be characterized as (1) Studies describing public health and laboratory research, including reviews, (2) Policy or social sciences research or analysis of Indian OH frameworks, and (3) Case studies of OH relevant programs or activities. Within these publications, we found that many of local OH activities occur through public health or laboratory research, often involving animal and human health researchers focusing on zoonotic diseases. We also found that outbreaks instigated local intersectoral coordination and collaboration, and there are some examples of community involvement or outreach in local OH activities.

Given the importance of OH action at the local level, it is encouraging to see research-related activities and implementation at this level. The documented research has largely focused on zoonotic diseases or AMR for public health surveillance. Intersectoral collaboration mostly happens between animal and human health researchers, though occasionally environmental sampling occurs, primarily for AMR studies. In general, we noted that the ecological sector was rarely engaged even when studies had a OH approach. Here, there is an opportunity for increased involvement of researchers outside of animal and public health fields, particularly within the environmental health, agricultural, and wildlife management sectors. As factors related to the transmission, emergence, and general etiology of zoonotic diseases are influenced by local environmental dynamics, this is a critical sector to engage both in research studies and in OH activity coordination.

A combination of survey and laboratory-based methods is often used in documented studies, including some incorporating economic analysis. Not only is multidisciplinary engagement critical for OH research, but similarly are multidisciplinary methods and input, which should be considered in future studies. The OH Consortium is an excellent initiative to enable intersectoral collaboration at multiple levels on OH activities through research; research collaborations established through other OH studies could support and integrate into this initiative.

There are several examples in which the veterinary community has supported diagnostics for diseases in human patients. The success of veterinary-supported COVID diagnostic testing and training demonstrates that veterinary health professionals can be important partners during human disease outbreaks, supporting the health system and workforce through laboratory diagnostics, clinical screening, and other logistics. Furthermore, they can also perform routine testing for zoonotic diseases in human samples outside of health emergencies. Cross-functional training in laboratory diagnostics could benefit both sectors in times of emergency.

Intersectoral collaboration is most common during disease outbreaks and stems from the urgent demand for action and human resources. A network analysis by Yasobant et al. shows that intersectoral interactions between actors at different levels (administrative, service providers, and community) of the health system in Ahmedabad, India are greater during an outbreak situation than during non-outbreak periods and they are directed in a top-down approach (22). Outside of outbreaks, there is little incentive for intersectoral collaboration. Yasobant et al. also found that solution-based OH initiatives or activities in India during outbreaks or health emergencies are robust and implemented at the grassroots level, but more level-based and integrated into third-party collaborations (example inter-ministerial OH task force) outside of outbreaks are necessary for a robust and resilient Indian health system (24).

Community-level engagement in or involvement in OH activities is lacking, as evidenced by our literature review. A qualitative and quantitative stakeholder analysis reveals that better hygiene and practices at the community level, communication at the grass root level, and development of OH Cell at the community level were among the top essential strategies for the operationalization of OH in the prevention and control of zoonotic diseases in Ahmedabad, India (32). Dasgupta et al. similarly emphasize the importance of strengthening community surveillance and community engagement through a bottom-to-top approach operationalized by community-based human and animal health workers, forest officers and rangers, farmers, and domestic animal owners across genders (21). Engaging community health workers may require incentives, as female (ASHA) and Male Multipurpose Health workers interviewed in Ahmedabad expressed low interest in serving as OH activists without financial compensation, recognition, and operational support or mandates (16).

While not documented in the published literature, the community outreach that veterinary institutions provide are vital. OH outreach to communities and improving awareness about zoonotic diseases, particularly in rural areas and farming communities, could have a tremendous impact on public engagement to address zoonotic diseases and prevention at the local level. Most Indian farmers are small livestock holders, raising animals for their sustainability. Their animals are usually kept in or near the same premises, increasing contact between animals and family members. Children also assist their parents in raising and caring for animals, activities that may expose them to zoonotic pathogens. However, there are gaps in zoonotic disease awareness in both adults (58–61) and children. A study conducted on school children (aged 11–16 years) in a semi-urban area of Karnataka showed that 31% of them (*n* = 320) were not aware of the initial steps to be taken after a dog bite, demonstrating the need to educate children about rabies, an endemic zoonotic disease in India (62). Therefore, zoonotic or OH outreach should be delivered to communities, including primary and secondary school education.

## Conclusion

The heterogeneity of India necessitates the OH framework that is robust at the local level. Factors that drive the emergence and persistence of zoonotic diseases, including high animal-human interactions, climate factors, etc., must be addressed at the grassroots level, the level at which they occur. From our review of the literature, it is apparent that OH-driven intersectoral coordination is built through research activities or ramped up *ad hoc* at the local level, for example, during outbreaks. This coordination should be formalized and sustained for ongoing, day-to-day activities like surveillance and disease notification that are important for detecting and responding to zoonotic diseases. Finally, the sustainability and success of OH frameworks depend upon the operationalization at the local level, including being conducted by local intersectoral teams with the knowledge and experience in the socio-cultural and political context of the specific area or community.

## Data availability statement

The original contributions presented in the study are included in the article/supplementary material, further inquiries can be directed to the corresponding author.

## Author contributions

JT, FP, BS, and ND conceptualized the manuscript topic. JT, FP, BS, ND, RS, JS, and AP contributed to the writing and editing of the manuscript. JT, RS, AP, JS, PK, BS, ND, and FP reviewed and extracted information from selected publications in literature review. RS, AP, JS, and BS contributed key inputs on local One Health activities occurring in India, including GADVASU and TANUVAS activities. JG and DG provided oversight and review of content and manuscript writing process. All authors contributed to the article and approved the submitted version.
